# The Effect of High Dose Radioiodine Therapy on Formation of Radiation Retinopathy During Thyroid Cancer Treatment

**DOI:** 10.4274/mirt.33042

**Published:** 2014-10-05

**Authors:** Tülay Kaçar Güveli, Sezer Özkan, Müge Öner Tamam, Ercan Uyanık, Nurcan Ediz, Mehmet Mülazımoğlu, Tevfik Özpaçacı

**Affiliations:** 1 Konya Training and Research Hospital, Clinic of Nuclear Medicine, Konya, Turkey; 2 Dünya Göz Hospital, Clinic of Ophtalmology, Samsun, Turkey; 3 Okmeydanı Training and Research Hospital, Clinic of Nuclear Medicine, İstanbul, Turkey

**Keywords:** Iodine-131, ablation, retinopathy, Thyroid neoplasms

## Abstract

**Objective:** Non-thyroidal complication of high-dose radioiodine therapy for thyroid carcinoma might cause salivary and lacrimal gland dysfunction, which may be transient or permanent in a dose-dependent manner. However, radiation retinopathy complicating 131I therapy, has not been previously well characterized. The aim of this study was to evaluate the extent of retinal damage among patients who had received high doses of radioiodine treatment.

**Methods:** Forty eyes of 20 patients (3 male, 17 female) who received 250-1000 mCi during 131I therapy and on ophthalmological follow up for a year after the last treatment were included in the study. Mean age of the study group was 50 years (range 25-70 years). In ophthalmologic examination, visual acuity was measured in order to determine visual loss. Intraocular pressure was measured in all the patients. Then lens examination was carried out with slit lamp biomicroscopy in order to investigate cataract or partial lens opacities. Fundus observation was carried out through the dilated pupil with slit lamp biomicroscopy using 90 D noncontact lens.

**Result:** The best corrected visual aquity with Snellen chart was found as 1.0 in 36 eyes (90%) and between 0.6 and 0.9 (10%) in 4 eyes (10%). At the biomicroscopic fundus examination, retinal hemorrhage consistent with radiation retinopathy, microaneurysm, microinfarction, edema or exudation, vitreus hemorrhage, partial or total optical disc pallor indicating papillopathy in the optic disc were not observed in any of the eyes.

**Conclusion:** This result indicates that there is not any significant correlation between repeated high-dose radioiodine therapy and radiation retinopathy in differentiated thyroid carcinomas. Even though there is not a significant restriction in use of higher doses of radioiodine therapy in differentiated thyroid carcinoma, more extensive studies are needed in order to obtain more accurate data on possible occurrence of retinopathy.

## Abstract (tr)

**Amaç:** Tiroid kanseri tedavisinde uygulanan yüksek doz radyoiyot tedavisi, doza bağımlı olarak geçici ya da kalıcı olabilen, tiroid dışı komplikasyon olarak tükrük ve gözyaşı bezleri fonksiyon bozukluğuna neden olabilir. Bununla birlikte I 131 komplikasyonları arasında radyasyon retinopatisi daha önce iyi karakterize edilmemiştir. Bu çalışmanın amacı yüksek doz radyoiyot tedavisi almış hastalarda retina hasarı oluşumunu değerlendirmekti.

**Yöntem:** Tedavisi sırasında 250-1000 mCi radyoiyot alan, son tedavisinin ardından 1 yıl oftalmolojik olarak izlenen 20 hastanın (3 erkek, 17 kadın) 40 gözü çalışmaya dahil edildi. Çalışma grubunun yaş ortalaması 50 (yaş aralığı 25-70) idi. Oftalmolojik incelemede ilk olarak görme kaybını belirlemek amacıyla görme keskinliği bakıldı. Tüm hastalarda göz içi basıncı ölçüldü. Sonrasında katarakt veya parsiyel lens opasitelerini araştırmak için yarık lamba biomikroskopisi ile lens muayenesi yapıldı. Doksan D nonkontakt lens kullanılarak pupiller dilate iken yarık lamba biomikroskopisinde fundusa bakıldı.

**Bulgular:** Snellen eşeli ile bakılan düzeltilmiş en iyi görme keskinliği 36 gözde (%90) 1,0 ve 4 gözde (%10) 0,6 ile 0,9 aralığındaydı. Biomikroskopik fundus muayenesinde gözlerin hiçbirinde radyasyon retinopatisi ile uyumlu olabilecek mikroanevrizma, mikroinfarkt, ödem veya eksüdasyon, optik diskte papillopatiyi gösteren parsiyel ya da total vitreus solukluğu, vitreus kanaması izlenmedi.

**Sonuç:** Bu sonuçlar diferansiye tiroid karsinomlarında tekrarlanan yüksek doz radyoiyot tedavisi ile radyasyon retinopatisi arasında anlamlı bir ilişki olmadığını göstermektedir. Diferansiye tiroid kanserlerinde yüksek dozlarda radyoiyot tedavisinde belirgin kısıtlama yoktur, retinopati oluşumunda daha detaylı bilgi sahibi olmak için daha kapsamlı çalışmalara ihtiyaç vardır.

## INTRODUCTION

Radioiodine (Radioiodine (RI) (iodine-131, or 131I)) is used for the ablation of postoperative residual thyroid tissue in differential thyroid carcinomas (1). Although serious acute complications are extremely rare following radioiodine treatment, several side effects could occur (2,3,4). Gastrointestinal complaints, salivary gland and lacrimal gland swelling with pain are mainly reported side effects, although there are no large prospective series on the side effects of radioiodine therapy in the literature. The current literature on this subject is mainly consists of retrospective studies and case reports (5).

Radiation retinopathy is an occlusive vasculopathy which begins in the late period following radiotherapy. It is generally seen after ionized radiation and 125I brachytherapy applied to the intraocular tumors, showing a slow progression and causing occlusion in retinal capillary, nonperfusion and large retinal vessels with neovascularization (6).

The literature about radiation-related retinopathy consists of the studies about external radiotherapy and brachytherapy. Clinical trials evaluating the patients for retinopathy following the treatment with radionuclides are quite limited. The aim of this study was to investigate whether high doses of radioiodine causes retinal damage during thyroid cancer treatment. Investigation of the clinical and pathological conditions regarding the ocular damage after external beam radiotherapy and brachytherapy may guide on the alterations after the treatment with radionuclides.

## MATERIALS AND METHODS

Between January 2000 and January 2007, 40 eyes of 20 patients who received 9250-37000 MBq (250-1000 mCi) during the therapy and on ophthalmological follow up for a year after the last treatment were included in the study. Three of the patients were males and 17 of them females aged between 25 and 70 (mean age:50±11). All patients were examined for detailed medical history, total blood count, routine biochemical tests, hypertension, hypercholesterolemia and diabetes. Patients with additional pathologies that might cause retinopathy were excluded.

Out of 20 patients, 17 were followed-up for papillary thyroid carcinoma and 3 for follicular thyroid carcinoma. Four of the patients had a history of radiotherapy received in the thyroid region and 2 due to metastasis to the lumbar vertebrae and pelvic bones. Characteristics of the patients are summarized in Table 1. 

Radioiodine ablation is performed four to six weeks after thyroid cancer surgery. After the operation patients are asked to avoid substances containing iodine. For postoperative ablation of thyroid bed remnants, activity in the range of 3700-7400 MBq (100-200 mCi) is typically administered. The patients, who developed recurrence during follow-up and received a total of 250-1000 mCi (mean dose 472 i±265) radioiodine with repeating doses, were included in the study. In addition, patients were questioned for the radiation damage to salivary and lacrimal glands and nasolacrimal duct that are the known side effects of radioiodine. 

In the ophthalmologic examination, first distance visual acuity rate was defined in order to determine visual loss. Patients were asked to read the Snellen chart with correction from a distance of 6 meters and the best corrected visual acuity (BCVA) was measured.

Intraocular pressure was measured in all patients with Goldman applanation tonometry. Proparacaine hydrochloride 0.5% was applied to all patients for the measurement. Then iris and iridocorneal angle were evaluated with slit lamp biomicroscope (Topcon SL-3C Tokyo, Japan) using Goldmann three-mirror contact lense. Iridocorneal angle was examined for neovascularization. Tropicamide drops of %1 was applied and the pupilla was dilated. Then lens examination was carried out with slit lamp biomicroscopy in order to investigate cataract or partial lens opacities.

Fundus observation was carried out on the dilated pupil with slit lamp biomicroscopy using 90 D noncontact lens. Especially symptoms including vitreus hemorrhage, partial or total optical disc pallor indicating papillopathy in the optic disc, retinal hemorrhage indicating retinopathy, microaneurysm, neovascularization, microinfarction, edema and exudation were examined. Colored images of fundus through dilated pupils were taken in all patients with digital retinal camera (Canon Inc Tokyo, Japan).

## RESULTS

The best corrected visual aquity with Snellen chart was found as 1.0 in 36 eyes (90%) and between 0.6 and 0.9 (10%) in 4 eyes (10%) There was pseudophakic posterior capsule opacification in two eyes and nuclear cataract in two eyes which did not interfere with fundus examination. Intraocular pressures were under 19 mmHg in all patients which was in the normal range. In the frontal segment examination carried out with slit lamp biomicroscopy, neovascularization in the iris and iridocorneal angle were not found in any of the patients.

There was pseudophakic posterior capsule opacification in one eye (5%) of two patients which has developed secondary to previous cataract surgery, reducing the best corrected acuity under 1.0. These patients later underwent neodymium-doped yttrium aluminum garnet (Nd:YAG) posterior capsulotomy procedure. The best corrected visual aquity reached to the level of 1.0 after the procedure. Nuclear cataract was found in 2 eyes (5%) of one patient, reducing the best corrected visual aquity under 1.0. After the surgeries , best corrected visual acuity of the patients reached to the level of 1.0 at the follow-ups.

At the biomicroscopic fundus examinations, retinal hemorrhage consistent with radiation retinopathy, microaneurysm, microinfarction, edema or exudation, vitreus hemorrhage, partial or total optical disc pallor indicating papillopathy in the optic disc were not observed in any eye. Two patients (10%) underwent dacryocystorhinostomy due to bilateral nasolacrimal duct obstruction developed following high dose RI therapy. 

**Figure 1:**
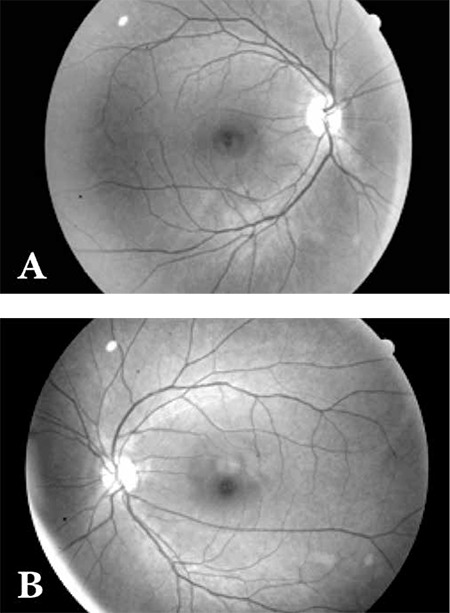
A 62 year old female patient had undergone total thyroidectomy and neck dissection due to follicular thyroid carcinoma 6 years ago. Shepostoperatively received 150mCi radioiodine therapy for ablation. The patient received a total of 900 mCi in repeated doses due to the development of lung and bone metastases. The patient has a complaint of lacrimal and salivary reduction. She was operated for nasolacrimal duct obstruction one year ago. Images taken with a digital retinal camera are presented above. At the fundus examinations, retinal hemorrhage, microaneurysm, microinfarct, edema or exudation were not observed.

Figure 1 shows images taken with a digital retinal camera in a patient.

## DISCUSSION

In the experimental trials, first alterations in the retina exposed to ionized radiation were detected to begin between the 12th and 24th months (7). Mainly capillary nonperfusion develops in the retinal capillary endothelial cells following focal loss. First damage occurs in the endothelial cells and pericytes. Early alterations seen in the retinal vessels include capillary dilatation, telangiectasia, microaneurysms and capillary occlusion. Then soft exudations are detected. Retinal edema and macular involvement may be seen. First symptoms are seen in deep small retinal vessels and later the large vessels are involved (8,9,10,11).

Following diffuse capillary occlusion and retinal ischemia, vitreous hemorrhage and retinal detachment as a result of retinal and/or disc neovascularization, atrophy of the retinal pigment epithelium, central retinal artery and central retinal vein obstructions have been reported (12,13).

Occlusive microangiopathy in radiation retinopathy is a progressive and slow process (8,14,15). Radiation retinopathy is observed after intraocular tumor irradiation (mainly uveal melanoma, less often retinoblastoma) or intracranial tumor radiotherapy. It is also seen following the irradiation for choroidal neovascularization due to age-related macular degeneration. Radiation doses and tumor location are mainly responsible for macular and/or optic nerve head vascular complications. Furthermore, choriocapillaris and main choroidal vessels may also be affected after the vascular and tissular retina complications (16).

In a study with 218 patients who received radiotherapy with a diagnosis of paramacular choroidal malignant melanoma, retinopathy symptoms were observed in 89% of the patients in a duration between 5 months and 15 years (mean 40 months) (17). Amoaku et al., reported that radiation retinopathy could be observed in an average period of 4.7 years after radiation therapy (range 1 to 8.5 years) (8). Whereas Brown et al. stated that radiation retinopathy may develop in 18.7 months (7-36 months) following teletherapy and 14.6 months (4-32 months) after brachytherapy (18). The cases included in that study were the patients in whom 1-10 years elapsed from the last radioiodine therapy. In this period, no pathological finding was found in these patients consistent with radiation retinopathy.

Incidence of radiation retinopathy depends on the total and fractionated dose applied (19). In the study by Brown et al., in the eyes undergone brachytherapy and developed radiation retinopathy, hard exudate was found in 85%,microaneurysms in 75%, intraretinal hemorrhages in 65%, retinal vascular telangiectasia in 35%, soft exudates in 30% and vascular encasement in 20%. Of the eyes undergone teletherapy and developed radiation retinopathy, hard exudate was found in 38%, microaneurysms in 81%, intrarenal hemorrhages in 88%, retinal vascular telangiectasia in 38%, soft exudates in 38% and vascular encasement in 25%. Of these eyes, macular edema was found in 87% at a 3-year follow-up and only 5% experienced spontaneous resolution (18).

The medium-long term side effects following radioiodine therapy were analyzed in an extensive study by Alexander et al. with 203 patients. The side effects occurred in the intermediate-term (up to 3 months) after therapy in 76.8% of the patients and in the long term (3 months and longer) in 61.1% of the patients. Cytoclastic side effects including sialoadenitis developed in 33% of the patients.

Looking to the other studies in the literature, late complications following RAI were rare and reported as aplastic anemia by 0.0%-1.2%, lung damage by 0.0%-0.4%, infertility by 0.4%-12%, temporary ovarian failure by 25% and persistent oligospermia by 0.4% (20,21,22,23).

Following radioiodine therapy, damage to lacrimal gland was reported in 42.9% of the patients after a period longer than 1 year. Complete xerostomia developed in 4.4% of the patients. Hematological abnormalities were observed in 9 patients, alopecia in 28.1% and chronic or repeating conjunctivitis in 22.7% of the patients, while 4 patients required dacryocystorhinostomy (20). There was not any study or case report in the literature about development of radiation retinopathy following radioiodine therapy. In our study, patients undergone high-dose radioactive iodine with repeated doses due to the recurrence and metastasis with a mean dose reached to 500 mCi (250-1000 mci) were included. Complaints of dry mouth, dysphagia and taste disorders were found in 10 patients (50%), while xerophthalmia was found in 2 (10%) and nasolacrimal duct obstruction in 2 (10%) patients. No clinical symptoms suggesting radiation retinopathy were found in any patients. We believed this was caused by the rapid excretion of radioiodine from the body in all the patients, because they had undergone thyroidectomy and neck dissection.

The most sensitive structure to ionized radiation in the eye is the lens. Cataract occurs in the lens at the end of a latent stage following the radiation. On the other hand, conjunctiva and cornea are moderately sensitive structures, while the retina and optic nerve are the most resistant structures in the eye (24). Epidemiological studies indicate an increase in incidence of the lens opacities at the doses under 1 Gy (25). Cataract stimulated by ionizing radiation (x-rays and gamma rays) is usually seen as posterior capsular or cortical cataract (26,27). Whereas, the cataract observed in one of our patients was of nuclear characteristics and evaluated as age-related senile cataract.

High-dose radioiodine therapy applied in thyroid carcinomas shows uptake in the head and neck region, suggesting partial exposure of the orbita and retina to the radiation. Based on this opinion, we evaluated the eyes of the patients exposed to high-dose radioiodine therapy. However, we did not find a significant correlation between repeated high-dose radioiodine therapy and radiation retinopathy in any of our patients. This might be a result of rapid excretion of radioiodine from the body in all the patients, because all cases had undergone thyroidectomy and neck dissection. The limitations of this study are the small group of patients and lack of control group. However, further extensive studies are needed on this subject.

## CONCLUSION

This study indicates that there is not any significant correlation between repeated high-dose radioiodine therapy and radiation retinopathy in differential thyroid carcinomas. Even though there is not any significant restriction in use of higher doses of 131I radioiodine therapy in differentiatedthyroid carcinomas, more extensive studies are needed in order to obtain more accurate data on possible occurrence of retinopathy in these cases.

**Conflicts of Interest**

There are no conflicts of interest.

## Figures and Tables

**Table 1:**
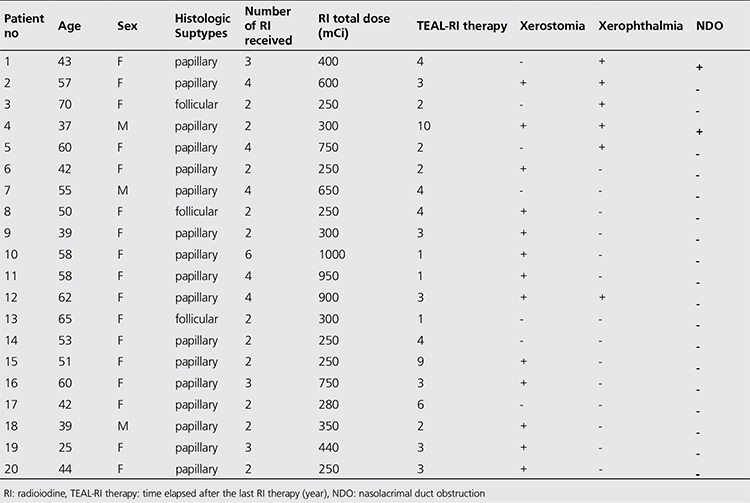
Patient characteristics

## References

[1] Samaan NA, Schultz PN, Hickey RC, Goepfert H, Haynie TP, Johnston DA, Ordonez NG (1992). The results of various modalities of treatment of well differentiated thyroid carcinomas: a retrospective review of 1599 patients. J Clin Endocrinol Metab..

[2] Markitziu A, Lustmann J, Uzieli B, Krausz Y, Chisin R (1993). Salivary and lacrimal gland involvement in a patient who had undergone a thyroidectomy and was treated with radioiodine for thyroid cancer. Oral Surg Oral Med Oral Pathol..

[3] Lin WY, Shen Y, Wang SJ (1996). Short term hazards of low dose radioiodine ablation therapy in postsurgical thyroid cancer patients. Clin Nucl Med..

[4] Malpani BL, Samuel AM, Ray S (1996). Quantification of salivary gland function in thyroid cancer patients treated with radioiodine. Int J Radiat Oncol Biol Phys.

[5] Sweeney DC, Johnston GS (1995). Radioiodine therapy for thyroid cancer. Endocrinol Metab Clin North Am.

[6] Maguire AM, Schachat AP (1994). Radiation retinopathy. In: Retina. 2nd ed. vol. 2.

[7] Irvine AR, Wood IS (1987). Radiation retinopathy as an experimental model for ischemic proliferative retinopathy and rubeosis iridis. Am J Ophthalmol.

[8] Amoaku WM, Archer DB (1990). Fluorescein angiographic features, natural course and treatment of radiation retinopathy. Eye.

[9] Chaudhuri PR, Austin DJ, Rosenthal AR (1981). Treatment of radiation retinopathy. Br J Ophthalmol.

[10] Gupta A, Dhawahir-Scala F, Smith A, Young L, Charles S (2007). Radiation retinopathy: case report and review. BMC Ophthalmol.

[11] Bianciotto C, Shields CL, Pirondini C, Mashayekhi A, Furuta M, Shields JA (2010). Proliferative radiation retinopathy after plaque radiotherapy for uveal melanoma. Ophthalmology.

[12] Hayreh SS (1970). Post-radiation retinopathy. A fluorescence fundus angiographic study.. Br J Ophthalmol.

[13] Boozalis GT, Schachat AP, Green WR (1987). Subretinal neovascularization from the retina in radiation Retinopathy. Retina.

[14] Archer DB, Amoaku WM, Gardiner TA (1991). Radiation retinopathy-clinical, histopathological, ultrastructural and experimental correlations. Eye.

[15] Wang W, Zhang XL, Wei SH (2012). Current research status on radiation retinopathy. Zhonghua Yan Ke Za Zhi.

[16] Grange JD (2001). Radiation-induced retinopathy. J Fr Ophtalmol.

[17] Guyer DR, Mukai S, Egan KM, Seddon JM, Walsh SM, Gragoudas ES (1992). Radiation maculopathy after proton beam irradiation for choroidal melanoma. Ophthalmology.

[18] Brown GC, Shields JA, Sanborn G, Augsburger JJ, Savino PJ, Schatz NJ (1982). Radiation retinopathy. Ophthalmology.

[19] Parsons JT, Bova FJ, Fitzgerald CR, Mendenhall WM, Million RR (1994). Radiation retinopathy after external-beam irradiation: analysis of time-dose factors. J Radiat Oncol Biol Phys.

[20] Alexander C, Bader JB, Schaefer A, Finke C, Kirsch CM (1998). Intermediate and Long-Term Side Effects of High-Dose Radioiodine Therapy for Thyroid Carcinoma. J Nucl.

[21] Brown AP, Greening WP, McCready VR, Shaw HJ, Harmer CL (1984). Radioiodine treatment of metastatic thyroid carcinoma: the Royal Marsden Hospital experience. Br J Radiol.

[22] Sarkar SD, Beierwaltes WH, Gill SP, Cowley BJ (1976). Subsequent fertility and birth and histories of children and adolescents treated with 131I for thyroid cancer. J Nuc Med.

[23] O’Doherty MJ, Nunan TO, Croft DN (1993). Radionuclides and therapy of thyroid cancer. Nuc Med Commun.

[24] Benson WE, Grande MG, Green WR, Heckenlively JR, Joeffe L, Murphy RP. (1990). Adverse effects of electromagnetic energy on the retina. In Retina and Vitreus; Section 4 Basic and Clin Sci Course of Am Academy of Ophtalmo.

[25] Rehani MM, Vano E, Ciraj-Bjelac O, Kleiman NJ (2011). Radiation and cataract. Radiat Prot Dosimetry.

[26] Lipman RM, Tripathi BJ, Tripathi RC (1988). Cataracts induced by microwave and ionizing radiation. Surv Ophthalmol.

[27] Merriam GR, Focht EF (1957). A clinical study of radiation cataracts and the relationship to dose. Am J Roentgenol.

